# Case Report: Tailored automatic speech recognition in global aphasia with dysarthria - a single case proof of concept

**DOI:** 10.3389/fresc.2026.1813312

**Published:** 2026-06-03

**Authors:** Davide Mulfari, Davide Cardile, Serena Campana, Loredana Voci, Carmelo Mario Vicario, Rocco Salvatore Calabrò, Francesco Corallo, Salvatore Mulfari, Francesco Tomaiuolo

**Affiliations:** 1 Assistive Technology for Special Needs Office, University of Messina, Messina, Italy; 2 IRCCS Centro Neurolesi Bonino-Pulejo, Messina, Italy; 3 Department of Health Sciences, “Magna Graecia” University of Catanzaro, viale Europa, Catanzaro, Italy; 4 Neurorehabilitation Unit, Auxilium Vitae Volterra, Volterra, Italy; 5 Department of Cognitive Sciences, Psychology, Education and Cultural Studies, University of Messina, Messina, Italy; 6 Department of Clinical and Experimental Medicine, University of Messina, Messina, Italy

**Keywords:** aphasia, assistive technology, automatic speech recognition, dysarthria, rehabilitation, speaker-dependent ASR, VIVOCA

## Abstract

**Background:**

Severe dysarthria and global aphasia drastically reduce speech intelligibility, confining communication to familiar partners. Automatic speech recognition (ASR) systems may show limited performance when processing such atypical speech.

**Objective:**

To determine whether a speaker-dependent Voice-Input Voice-Output Communication Aid (VIVOCA) embedded in the CapisciAMe app can decode the speech of a person with severe dysarthria and aphasia more accurately than rehabilitation professionals human listeners (RPHL).

**Methods:**

We conducted a single-case proof-of-concept study. A 34-year-old woman, 15 years post-stroke, recorded 1,120 utterances of 13 target-words across five prompting modalities. A compact convolutional neural network (cnn-trad-fpool3) was trained on these samples and evaluated on an independent set of 936 utterances. Intelligibility was benchmarked against 12 RPHL familiar with the patient. The primary outcome was word-level accuracy.

**Results:**

The tailored ASR achieved 72.65 % accuracy, outperforming familiar RPHL (mean = 56.75 %, SD = 12.91).

**Conclusions:**

A personalized ASR system can exceed the intelligibility of human listeners for profoundly disordered speech, supporting its use as an assistive communication technology.

## 1 Introduction

Dysarthria is a motor speech disorder arising from impaired neuromuscular control (1). Although various types exist (2), these speech impairments can result from damage at various locations of the neuroaxis, including: the motor cortex, basal ganglia, brainstem nuclei, cerebellum, or descending corticobulbar pathways, often affecting cranial nerves V, VII, IX, X, and XII which are critical for respiration, articulatory precision, prosody and speech timing (3, 4). Beyond motor impairment, individuals with dysarthria frequently face significant psychosocial challenges, including social withdrawal, depression, and reduced quality of life, especially when intelligibility is severely compromised  (5, 6).

These effects are even more pronounced when dysarthria co-occurs with global aphasia after large left-hemispheric lesions a condition marked by profound deficits in expressive and receptive language.

Following large left-hemispheric strokes, especially involving the perisylvian cortex and deep white matter, verbal communication becomes drastically limited, and the patient may rely entirely on familiar partners for interaction  (7, 8).

Recent advances in machine learning have rekindled interest in automatic speech recognition (ASR) as a tool for augmentative and alternative communication (AAC) (9).

In clinical populations with atypical speech, such as post-stroke dysarthria, generic ASR systems often fail due to mismatched acoustic models and high speech variability  (10, 11). However, speaker-dependent systems (models trained exclusively on a single individual's voice) offer a promising solution by adapting to the unique phonetic and articulatory patterns of the user (12). These systems have demonstrated improved word-level accuracy even in the presence of severe motor speech impairments  (13).

The CapisciAMe app was developed in Italy to explore personalized ASR vocabularies for people with motor speech disorders (14). We report a single-case evaluation of a CapisciAMe VIVOCA configured for an adult patient with global aphasia and severe dysarthria. System performance (15) was compared with that of rehabilitation professionals human listeners (RPHL) who routinely communicate with her. This case adds to emerging evidence supporting customized ASR systems in augmenting communication for motor speech disorders.

## 2 Method

### 2.1 Case description

**Figure 1:**
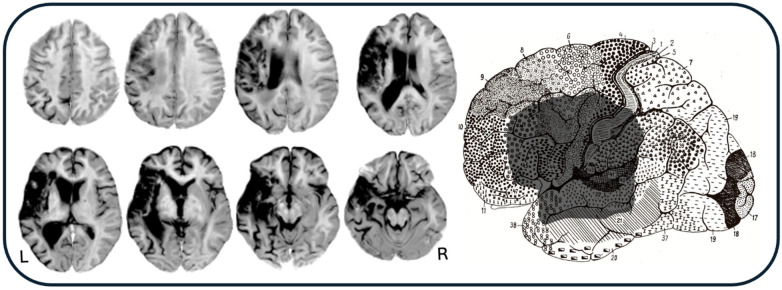
Axial brain MRI scans highlight an extensive neuroanatomical disruption affecting the left opercular region of the patient. The figure shows T2-weighted MRI with reversed grayscale contrast performed 18 months from the cerebrovascular accident. The first image series from left to right shows progressive slices of the brain at different levels from top to bottom. The localization and extent of the lesion was depicted on the left hemisphere with reference to Broadman's map areas. The most posterior part of the superior and middle temporal gyri was partially spared as well as the angular gyrus and part of the supramarginal gyrus.

The patient was a 34-year-old right-handed adult (education: 12 years) who sustained a massive left-hemispheric hemorrhage when the patient was 19 years old. Surgical excision of an arteriovenous malformation and occlusion of the left middle cerebral artery were performed. Follow-up MRI at 18 months revealed an extensive lesion encompassing the opercular, temporo-fronto-parietal and putaminal regions (Figure 1). The patient presented with right hemiparesis, global aphasia and dysarthria. Despite minimal verbal output, non-verbal reasoning was preserved (Raven's = 43/60). Language abilities were evaluated using the Esame Neuropsicologico per l'Afasia. (E.N.P.A.; Capasso & Miceli, 2001), at two time-points at 14 and 28 months form injury. This longitudinal clinical profile underscores a marked recovery in cognitive and communicative intentionality, despite persistent severe verbal limitations. The preserved reasoning skills and enhanced social interaction provide a strong foundation for introducing assistance. A more detailed clinical and linguistic profile is available in Supplementary Appendix 1.

### 2.2 Vocabulary and prompting modalities

A 13-word Italian vocabulary set (okay: okay, entra: enter, dietro: back, avanti: forward, volume: volume, chiudi: close, sinistra: left, destra: right, stop: stop, esci: exit, su: up, giù: down, apri: open) previously validated in a home-automation project was adopted  (14), keeping the vocabulary small enough to train a reliable speaker-dependent model.

Patient speech production was elicited using the following five prompting modalities. More specifically, the five modalities were selected to engage partially distinct mechanisms of word access: combined audiovisual cueing “Text-to-caption and Text-to-speech” (M1), auditory-only prompting “Text-to-speech only” (M2), picture-based semantic access “Pictures only” (M3), orthographic cueing “Text caption only” (M4), and contrast-based semantic facilitation “Antonym-Based Prompting” (M5). This multimodal elicitation strategy was intended to accommodate the heterogeneity of preserved and impaired language processes in global aphasia, while also generating a more diverse and clinically realistic speech sample for speaker-dependent ASR training.

### 2.3 Data collection and model training

Over one month, the patient completed 33 recording sessions (approximately three times per day, three days per week) in a quiet therapy room using a headset microphone (44.1 kHz, 16-bit). For each item of the 13-word Italian vocabulary, a single target-word was elicited using one of the five prompting modalities described in Table 1. The corpus was partitioned using a session-based split to prevent temporal leakage: all utterances recorded in November 2022 were reserved as the held-out test set (936 utterances; M1 = 94, M2 = 208, M3 = 97, M4 = 165, M5 = 372), which remained completely unseen throughout training and model selection. The remaining pre-November recordings, comprising speech from the target speaker and 69 additional native Italian speakers with dysarthria, were randomly split at the utterance level into training (95%) and validation (5%) sets, yielding 21,947 and 1,162 utterances respectively. A stratified sampling strategy was applied to preserve the relative frequency of each target-word across all three subsets, avoiding class imbalance. The validation set was used exclusively for hyperparameter tuning and early stopping monitoring, with no overlap with the test set.

**Table 1:** Summary of the five prompting modalities used to train the patient's personalized CapisciAMe ASR system.

Modality	Test	Task
M1	Text-to-caption and Text-to-speech	Word was both displayed on the screen and played aloud using text-to-speech synthesis
M2	Text-to-speech only	The word delivered exclusively through synthesized audio without any visual cue
M3	Pictures only	An image representing the word was shown to prompt a naming response
M4	Text caption only	The word appeared on the screen without accompanying audio
M5	Antonym-Based Prompting	A semantically opposite word was presented, either visually or orally, to prompt production of the target word through contrast.

All recordings were resampled to 16 kHz, converted to mono, and amplitude-normalized to a fixed peak level. Leading and trailing silences were removed via energy-based voice activity detection (VAD; threshold = −40 dB, minimum speech duration = 200 ms), and background noise was attenuated using spectral gating (*noisereduce* library). No data augmentation was applied. Acoustic features were extracted as 39-dimensional Mel-Frequency Cepstral Coefficients (MFCCs), including delta and delta-delta derivatives, arranged as 2D time-frequency representations and zero-padded or truncated to a fixed length to ensure uniform input dimensions.

The cnn-trad-fpool3 network (16) was trained with the Adam optimizer (learning rate = 1 × 10⁻^4^, batch size = 64) for up to 40 epochs, with early stopping triggered after 3 consecutive non-improving validation steps. All random seeds were fixed to ensure reproducibility. Results are reported from a single training run.

Full details of the architecture and feature extraction procedure are provided in Supplementary Section 2.2 of the Supplementary Appendix.

### 2.4 Human speech intelligibility benchmark

To compare ASR performance with human perception, twelve Italian RPHL (6 female, 6 male; mean age = 38.92; DS 9.35), from a post-acute stroke rehabilitation unit were recruited to assess speech intelligibility. All RPHL had undergone routine occupational health evaluation for work fitness, and no auditory deficits had been reported by the occupational physician for any of them. This group was intentionally selected as a stringent human benchmark, as their daily clinical exposure to patients with impaired speech and language likely made them more sensitive than naïve listeners to degraded post-stroke speech. By comparing the ASR system against listeners with enhanced perceptual familiarity, we aimed to test the system under demanding conditions and to provide a more conservative estimate of its performance. To minimize bias, speech-language therapists directly involved in the patient's treatment were excluded from the listening task. All participants provided informed consent.

Each RPHL was presented with 26 utterances, comprising two different recorded exemplars for each of the 13 target-words. For every target-word, the two stimuli corresponded to distinct verbal productions drawn from the patient's speech recordings, thereby avoiding repeated presentation of the same token. The listening task was conducted in a quiet room, and the recordings were delivered through loudspeakers. Stimuli were presented without visual or contextual cues, and listeners were instructed to report freely the word they believed the patient intended to say, without being provided with response alternatives (open-set paradigm). Response accuracy was recorded by a neuropsychologist serving as the examiner on a precompiled scoring sheet. Accuracy was defined as the proportion of correctly identified target-words over the total number of utterances presented.

### 2.5 Outcome measures and data analysis

Given the single-case design of this study, all statistical analyses are intended as descriptive and exploratory tools to characterize performance differences, rather than as confirmatory tests of generalizable hypotheses. The primary outcome was word-level classification accuracy, defined as the proportion of correctly identified target-words. ASR accuracy was computed as a single proportion with Wilson score 95% confidence interval (CI). Wilson score intervals were applied to the ASR accuracy given its binomial nature (correct/incorrect per item), as this method provides reliable coverage even at extreme proportions. For the RPHL group, individual listener accuracy was computed for each of the 12 participants as the proportion of correctly identified utterances out of 26. Group-level uncertainty was estimated via a bootstrap 95% CI based on 10,000 resampling iterations from the 12 individual scores, treating the listener as the unit of analysis to appropriately account for the non-independence of responses nested within raters.

To test whether ASR accuracy significantly exceeded human listener performance, a one-sample t-test was performed using the 12 individual listener accuracy scores as observations and the ASR accuracy as the reference value. A non-parametric Wilcoxon signed-rank test was additionally computed to verify robustness against violations of normality assumptions given the small sample size (*n* = 12). Inter-rater reliability among listeners was assessed using the intraclass correlation coefficient [ICC(2,1), two-way random effects, single measures], computed across the 12 listeners and 26 utterances. Per-target-word listener accuracy was computed descriptively as the proportion of correct recognitions across all listeners (24 observations per target-word: 2 utterances × 12 listeners). All analyses were conducted in Python (SciPy 1.x; NumPy 1.x).

## 3 Results

The tailored ASR correctly identified 680 of 936 test utterances, yielding an overall word-level accuracy of 72.65% (95% CI 69.71–75.41). Per-modality accuracy ranged from 54.6% (M3, picture-only prompting) to 84.1% (M2, audio-only prompting), as reported in Table 2.

**Table 2:** ASR target-word recognition accuracy (WRA) across the five prompting modalities.

Modality	Test Utterances	WRA (%)
M1	94	81.9
M2	208	84.1
M3	97	54.6
M4	165	75.2
M5	372	67.7
Total	936	72.65

Individual listener accuracy ranged from 38.5% to 73.1% (mean = 56.75%, SD = 12.91%; To estimate the 95% confidence interval for listener accuracy, we used bootstrap resampling, given the small sample size and the absence of assumptions regarding the underlying distribution. The bootstrap-derived 95% confidence interval was 49.38% to 63.48%, indicating substantial variability across evaluators. Inter-rater reliability was poor [ICC(2,1) = 0.367], further highlighting the inconsistency of human perceptual judgments for this patient's severely dysarthric speech. Per target-word analysis revealed substantial variability in listener accuracy across the 13 target-words, ranging from 100.0% for Ok to 16.7% for “apri” (open), with phonologically less salient or more ambiguous items consistently yielding lower recognition rates (Table 3).

**Table 3:** Target-word recognition accuracy across 12 rehabilitation professionals' human listeners (2 utterances × 12 listeners = 24 observations per-word).

Target-Word	Correct (n/24)	Listener accuracy (%)
ok (*ok)*	24/24	100.0
entra (*enter*)	22/24	91.7
dietro (*back*)	20/24	83.3
avanti (*forward*)	20/24	83.3
volume (*volume*)	19/24	79.2
chiudi (*close*)	17/24	70.8
sinistra (*left*)	16/24	66.7
destra (*right*)	9/24	37.5
stop (*stop*)	7/24	29.2
esci (*exit*)	7/24	29.2
su (*up*)	6/24	25.0
giù (*down*)	6/24	25.0
apri (*open*)	4/24	16.7

**Figure 2:**
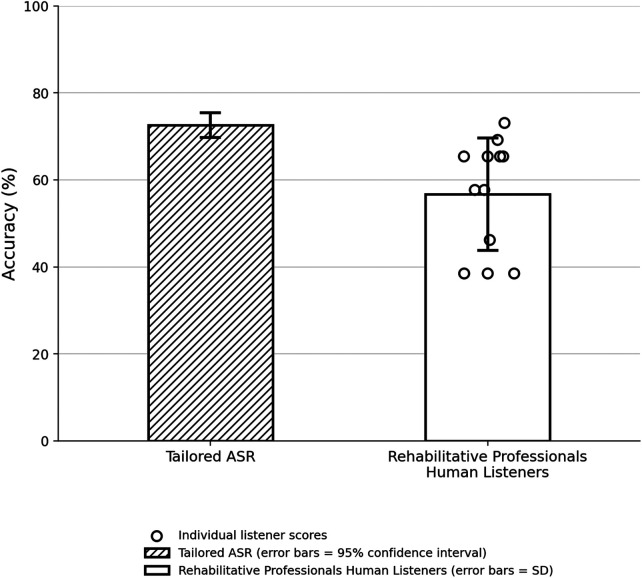
Mean word-level accuracy for the tailored ASR system and the human listener control group. For the ASR, error bars represent 95% confidence intervals (Wilson score interval). For the human listener group, error bars represent the standard deviation (SD); black dots indicate individual listener scores (*n* = 12). The ASR correctly recognized 680 of 936 utterances (72.65%, 95% CI 69.71–75.41); the listener group mean was 56.75% (SD = 12.91%).

The ASR system's accuracy (72.65%) exceeded the Bootstrap 95% CI for the listener group (49.38–63.48%), indicating a non-overlapping advantage. Treating the listener as the unit of analysis (*n* = 12), a one-sample *t*-test confirmed that the ASR accuracy was significantly higher than the listener distribution [t(11) = −4.27, *p* = 0.001]. A non-parametric Wilcoxon signed-rank test yielded a convergent result [W = 1.0, *p* = 0.001], confirming robustness irrespective of distributional assumptions. The absolute advantage of the ASR over the listener mean was 15.9 percentage points. Figure 2 displays mean accuracy for both groups with 95% CIs and individual listener scores.

A sensitivity analysis was conducted to determine the minimum effect size detectable with the available listener sample (*n* = 12, *α* = .05, one-tailed). With 80% power, the minimum detectable effect size was *d* = 0.77, corresponding to a difference of approximately 9.9 percentage points: with 95% power, *d* = 1.02 (13.1 percentage points). The observed difference of 15.9 percentage points (*d* = 1.23) substantially exceeds both thresholds, confirming that the sample was adequately sensitive to detect an effect of the magnitude observed.

## 4 Discussion

This single-case study shows that a personalized VIVOCA integrated into the CapisciAMe app decodes profoundly disordered speech more accurately than RPHL. Across an independent test set, the tailored recognizer consistently outperformed the RPHL. For this speaker with severe dysarthria and global aphasia, a speaker-dependent ASR therefore functions as an amplifier of intelligibility, supporting more reliable communication in everyday contexts (17, 18).

Beyond the motor speech impairment, this case is marked by global aphasia, which severely constrains linguistic formulation and comprehension. Within this context, the observed benefit of a personalized VIVOCA reflects not only better decoding of highly atypical articulatory patterns but also a pragmatic workaround for the lexical–semantic bottlenecks typical of global aphasia. By centering communication on a compact, functionally salient vocabulary, the system lowers linguistic load and supports reliable expression of high-priority intents, an approach consistent with frameworks in augmentative and alternative communication that prioritize communicative competence and participation over restoration of normal language. Evidence from picture-based, computer-supported programs for global aphasia shows that several individuals convey more information with aided communication than with natural communication alone, underscoring the practical value of such supports (19).

Notwithstanding these promising findings, this study employs a single-case design, centered on a person with language and motor speech impairments. Although the personalized ASR system yielded promising outcomes, the results are not immediately generalizable to broader populations with more variable profiles. The limited training vocabulary also constrains the communicative breadth of the system. Additionally, the human benchmark consisted of rehabilitation professionals with sustained clinical exposure to the patient's communicative style, constituting a familiar-listener baseline rather than a general one. From a methodological standpoint, this choice is ecologically valid since RPHL represent the patient's primary real-world communicative partners. This implies that the reported advantage of the ASR system should not be generalized to the broader population of human listeners. It is reasonable to expect that unfamiliar listeners, lacking prior exposure to the patient's idiosyncratic articulatory patterns, would perform at substantially lower accuracy levels, which would further increase the relative advantage of the personalized ASR. Future studies should include both familiar and unfamiliar listener groups to establish a more complete intelligibility profile and to better quantify the clinical utility of speaker-dependent ASR across different communicative contexts. These considerations align with broader observations that generic recognizers struggle with dysarthric speech, while speaker-specific adaptation consistently narrows the gap (20), a pattern that further motivates expanding personalized ASR evaluations to more heterogeneous listener and speaker populations.

From a statistical standpoint, the inferential comparison between ASR and RPHL was conducted treating the RPHL as the unit of analysis (*n* = 12), thereby addressing the non-independence of responses nested within raters. The poor inter-rater agreement observed (ICC = 0.367) underscores the inherent variability of human intelligibility judgements for profoundly disordered speech, further supporting the consistency advantage of a personalized ASR system. A residual limitation is the absence of a prospectively defined shared evaluation subset: the specific utterances presented to listeners were not prospectively logged, precluding a fully paired item-level comparison between ASR and human responses. This will be prioritized in future work.

Future directions should include expanding the system's lexicon to support more flexible and spontaneous communication. Comparative evaluations against strong, general-purpose recognizers and against unfamiliar listeners would help establish more robust performance benchmarks. In parallel, advances in large-scale speech and language modeling, such as weakly supervised models trained on hundreds of thousands of hours, offer practical routes to improved robustness when combined with speaker-specific adaptation; personalization with limited data has already shown large error-rate reductions for atypical speech (21).

Finally, as proposed by Tröger et al. (22), future evaluations should pair recognition accuracy with person-centered outcomes such as daily use, communicative effectiveness, caregiver burden, and satisfaction and include validated digital indicators of intelligibility. One practical option is an automated speech-intelligibility score, developed and validated across several motor-speech disorders and languages, which provides a scalable, objective outcome that complements clinician ratings and supports sensitive tracking over time.

In conclusion, customized ASR systems represent a transformative assistive technology for individuals with severe dysarthria and aphasia, restoring aspects of communicative independence that may be unattainable through unaided human interaction. The CapisciAMe VIVOCA exemplifies how user-tailored technology can enhance autonomy, social participation, and overall quality of life, and the trajectory from early voice-input/voice-output systems to large-scale personalization efforts supports continued development in this direction (23).

## Data Availability

The raw data supporting the conclusions of this article will be made available by the authors, upon reasonable request.
