# Supplementary material for: Case Report: Tailored automatic speech recognition in global aphasia with dysarthria - a single case proof of concept
Source: Front Rehabil Sci. 2026 Jun 3;7:1813312. doi: 10.3389/fresc.2026.1813312 (PMC13271983; doi:10.3389/fresc.2026.1813312)
Supplement: Supplementary file 1 [file Supplementaryfile1.docx]

# Supplementary Material

**Tailored Automatic Speech Recognition for a Patient with Global Aphasia and Dysarthria: A Single‑Case Proof‑of‑Concept Study**

Davide Mulfari, Davide Cardile*, Serena Campana, Loredana Voci, Carmelo Mario Vicario, Rocco Salvatore Calabrò, Francesco Corallo, Salvatore Mulfari, Francesco Tomaiuolo.

## 2.1 Case Report

The patient is a 34-year-old right-handed adult with 12 years of formal education. At the age of 19, the patient suffered a massive left-hemispheric intraparenchymal hemorrhage. Emergency neurosurgical intervention involved the excision of an arteriovenous malformation and the occlusion of the left middle cerebral artery. CT brain scan conducted 18 months later revealed an extensive ischemic lesion affecting the opercular region and extending through the left temporo-fronto-parietal cortex to the putamen (Figure 1).
Seventy-seven days after surgery, the patient was admitted to inpatient rehabilitation in a non-responsive state. Over the following weeks, the patient regained consciousness and emerged from a minimally conscious state. A neuropsychological assessment conducted 11 months post-onset documented right hemiplegia, left upper limb hemiparesis, and severely reduced verbal output. Nonetheless, non-verbal cognitive abilities appeared relatively preserved: the patient achieved a raw score of 43/60 on the Raven’s Progressive Matrices 38 (cut-off = 21), indicating intact abstract reasoning. Performance on visual matching and color recognition tasks was also adequate. During this period, communication was limited to affective expressions (e.g., crying) and head movements to signal yes/no responses.

By 14 months post-injury, the patient demonstrated improved contextual comprehension and began engaging more effectively with the environment. The patient was able to produce several meaningful bi-syllabic words and appropriate vocalizations for basic needs (e.g., indicating hunger or discomfort). Simple word repetition became possible, and gestures were used to support verbal attempts. However, articulation remained imprecise, spontaneous speech was minimal, and writing abilities were restricted to copying; spontaneous naming was absent. The patient continued to require full assistance with activities of daily living.

At 28 months post-onset, follow-up examination revealed right homonymous hemianopia and mild bucco-facial apraxia. Communicative intentionality and pragmatic use of available verbal and non-verbal resources had substantially improved. The patient interacted more effectively with caregivers and therapists, suggesting emerging cognitive-linguistic integration and increased psychosocial engagement.

At the time of study inclusion (15 years post-onset) the patient still presented with global aphasia, severe spastic dysarthria, and right hemiparesis. Expressive language remained limited to isolated words or short telegraphic utterances. Speech intelligibility was extremely reduced, especially for unfamiliar listeners.

Language abilities were evaluated at two time points at 14 and 28 months from injury (Table 1) using the Esame Neuropsicologico per l'Afasia (E.N.P.A., Capasso & Miceli, 2001).

*Table 1: Comprehension scores collected at two time points: at 14 and 28 months from injury. Raw and adjusted scores are reported for auditory and visual word comprehension, and auditory and visual sentence comprehension.*

| **E.N.P.A.** | | | **N** | **Raw score**  (14 months post injury)) | ^**Adjusted score** (14 months post injury) | **Raw score**  (28 months post injury) | **^Adjusted score** (28 months post injury)) | **Cut-Off adjusted score ^** |
| --- | --- | --- | --- | --- | --- | --- | --- | --- |
| Comprehension | Word | auditory | *20* | **13** | **12.4*** | *18* | *17.4** | **18.4** |
|  |  | visual | *20* | **13** | **12.3*** | *12* | *11.3** | **17.0** |
|  | Sentences | auditory | *14* | **8** | **7.5*** | *9* | *8.5** | **11.6** |
|  |  | visual | *14* | **8** | **7.6*** | *12* | *11.6* | **11.3** |

*^ Adjusted score for age <45 and schooling of 9-13 years; * score below the Cut-Off.*

Word-level comprehension remained impaired in both auditory and visual modalities, with more pronounced deficits in the visual domain. In contrast, sentence comprehension showed relative improvement when visual cues were present, pointing to preserved syntactic processing supported by contextual information.

This longitudinal clinical profile underscores a marked recovery in cognitive and communicative intentionality, despite persistent severe verbal limitations. The preserved reasoning skills and enhanced social interaction provide a strong foundation for introducing assistive communication technologies, such as the personalized ASR-based VIVOCA system, tailored to the patient’s unique communicative profile.

## 2.2 ASR System and CapisciAMe App

The CapisciAMe ASR system is built on the cnn-trad-fpool3 architecture, a compact convolutional network designed for small-footprint taget-word spotting. The model takes as input 39-dimensional MFCC feature vectors (comprising 13 base coefficients plus delta and delta-delta derivatives, extracted with a 25 ms Hamming window, 10 ms hop size, and a 40-channel Mel filterbank, with utterance-level CMVN normalization) arranged as 2D time–frequency representations of fixed length. Two convolutional layers sequentially process these representations: the first applies 20 filters of size 8×8 to capture broad spectro-temporal patterns, and the second applies 64 filters of size 1×3 for finer temporal refinement. A max-pooling layer (filter size 1×3) follows, reducing dimensionality and mitigating overfitting. A fully connected layer then maps the extracted representations to a high-level feature space, and a final softmax layer outputs classification probabilities over the 13 target-words. By design, the architecture's limited number of layers and parameters makes it well suited for deployment on resource-constrained devices such as single-board computers or smartphones. Training was performed using the Adam optimizer (learning rate = 1×10⁻⁴, batch size = 64) for up to 40 epochs, with early stopping triggered after 3 consecutive non-improving validation steps; all random seeds were fixed to ensure reproducibility. The system was trained on the patient's recordings to adapt to her unique articulatory patterns.
